# High NE dose trajectory is associated with new onset of acute kidney injury patients: A group-based trajectory modeling analysis

**DOI:** 10.1371/journal.pone.0323431

**Published:** 2025-05-13

**Authors:** Jinfeng Yang, Yangzi Liujiao, Xijing Zhang, Jiong Xiong, Fengming Wang, Feng Shen

**Affiliations:** 1 Department of Critical Care Medicine, the Affiliated Hospital of Guizhou Medical University, Guiyang, China; 2 School of Clinical Medicine, Guizhou Medical University, Guiyang, Guizhou Province, China; Azienda Ospedaliero Universitaria Careggi, ITALY

## Abstract

**Background:**

Norepinephrine (NE) is a first line and effective vasopressor for septic shock management, but its impact on newonset acute kidney injury (AKI) in those patients remains controversial. This study sought to investigate the relationship between norepinephrine dose trajectories and the new occurrence of AKI during the management of septic shock by using NE.

**Methods:**

A retrospective cohort study was conducted using the MIMIC-IV database, which includes 3,462 patients diagnosed with septic shock during the initial 96 hours following their admission to the ICU. The unique patterns of trajectory analysis of NE were characterized by using group-based trajectory modeling (GBTM) during the initial four days of ICU admission. We employed multivariable logistic regression analysis and subgroup analysis to evaluate the association between NE dose trajectories and new-onset AKI in patients with septic shock.

**Results:**

Three NE dose trajectories were identified: low NE dose (47.3%), middle NE dose (41.5%), and high NE dose (11.2%). The high NE dose trajectory had significantly higher risks for new onset of AKI (OR 2.39, 95% CI 1.43–3.99), MAKE-30 (OR 3.82, 95% CI 2.97–4.91), and for 28-day mortality (HR 2.01, 95% CI 1.70–2.37) compared to the low NE dose trajectory. Despite over 90% of patients in the middle NE dose trajectory developing AKI, patients in this trajectory exhibited a lower risk of MAKE-30 and 28-day mortality. After comprehensive adjustment for demographic characteristics, comorbidities, acute physiological status, laboratory indicators, and fluid management, high NE dose trajectory remained independently associated with increased risk of new-onset AKI (OR 1.39, 95% CI 1.04–1.86, P = 0.024), this association persisted across multiple subgroup analyses.

**Conclusion:**

During the management of septic shock, high dose of NE trajectory was associated with high likelihood of new onset of AKI, high possibility of MAKE-30 and high 28-day mortality in patients with septic shock. High NE dose trajectory serves as an independent predictor for assessing the risk of new-onset AKI in patients with septic shock.

## Introduction

Septic shock is a severe complication of sepsis, commonly observed in critically ill patients and is acknowledged as a major factor in the occurrence of acute kidney injury (AKI) within this population [1,2], with the mortality rate associated with septic shock exceeding 40% [3].

The profound hemodynamic instability caused by septic shock necessitates the administration of vasopressors following fluid resuscitation for effective management [4]. Among these agents, norepinephrine (NE) is widely endorsed as the primary vasoactive medication for managing septic shock [5]. Nevertheless, several studies have indicated that NE treatment is linked to adverse effects, such as a heightened risk of mortality and organ failure in critically ill patients [6,7].

Furthermore, the effects of NE and other vasoactive drugs on sepsis-associated AKI remain controversial. Previous studies have demonstrated that early administration of NE in patients with hypotensive sepsis can enhance cardiac output (CO) and improve microcirculatory function [8]. However, some patients may develop resistance to NE, resulting in refractory hypotension and progression to septic shock. Additionally, prolonged use of high-dose NE has been closely associated with increased mortality and impaired organ function [9]. These prior studies considered only a single point measurement of NE dosage. Few investigations have examined the longitudinal dynamics of NE dosing in patients experiencing septic shock and its correlation with the risk of AKI over time.

To address this gap, the present study performed a retrospective analysis of data from the Medical Information Mart for Intensive Care IV (MIMIC-IV) database to explore the potential correlation between NE dosage trajectories and the incidence of new-onset AKI in septic shock patients within critical care settings. We hypothesize that over the subsequent four days, NE dosage will exhibit distinct trajectories, which may be associated with varying incidence of AKI, renal adverse events, and 28-day mortality among patients suffering from septic shock.

## Methods

### Data sources

The MIMIC-IV database was employed, representing a comprehensive and anonymized compilation of electronic health records obtained from a single medical institution. This database includes patient data from Beth Israel Deaconess Medical Center, covering hospitalizations between 2008 and 2019 [10]. The MIMIC-IV database was utilized to investigate critically ill patients who experienced septic shock during the initial 96 hours following their admission to the intensive care unit (ICU).

### Feature extraction

A comprehensive array of data was collected, encompassing patient demographics, underlying comorbidities, laboratory results, mechanical ventilation usage, fluid balance, and total fluid load. Clinical and laboratory data were employed to assess disease severity, including the Sequential Organ Failure Assessment (SOFA) score [11]. In accordance with published guidelines, variables with more than 40% missing data were excluded from the analysis [12]. To address the issue of missing data, we applied the multivariate imputation by chained equations (MICE) method in R, utilizing predictive mean matching to ensure robust estimations [13].

### Definition

The NE dosage was determined using the methodology established by Jentzes et al.[14]. Based on the equivalent dosage conversion formula for vasoactive drugs, the corresponding dosages of various catecholamines to NE are as follows: 0.1 µg/kg/min epinephrine is equivalent to 0.1 µg/kg/min of NE, 15 µg/kg/min of dopamine corresponds to 0.1 µg/kg/min of NE; 1 µg/kg/min of phenylephrine is equivalent to 0.1 µg/kg/min of NE; and 0.04 µg/kg/min of vasopressin is also equivalent to 0.1 µg/kg/min of NE. This formula encompasses a range of standard vasoactive drugs. Instead of merely norepinephrine, this method standardizes the effects of various vasoactive drugs to an equivalent norepinephrine dose. Although this reflects the combined effect of multiple vasoactive drugs, in our study population, most patients predominantly received norepinephrine therapy, with other drugs used as adjuncts only when clinically necessary. However, in clinical practice, patients may not receive all medications listed in the formula and may also use some vasoactive drugs that are not included therein. Therefore, during data analysis, only the drugs administered to the patient that are specified in the formula should be considered, while any unlisted drugs should be excluded from the calculation. This approach ensures accuracy in data analysis, allowing for an accurate reflection of the overall efficacy of the administered vasoactive drug regimen through calculated NE equivalent doses.

Sepsis was characterized by a decline of life-threatening organ function resulting from infection within 48 hours following the patient’s admission to the critical care unit or during their stay therein. Septic shock was defined as persistent hypotension due to sepsis requiring vasoactive drugs for maintaining mean arterial pressure (MAP) above 65 mmHg despite adequate volume resuscitation, with serum lactate concentration remaining above 2 mmol/L [15].

AKI and its severity were classified according to the Kidney Disease Improving Global Outcomes (KDIGO) guidelines [16]. To assess kidney function, we utilized estimated baseline serum creatinine (SCr) and the lowest SCr value recorded upon ICU admission, selecting the lower value as our reference point [17].

Fluid balance (FB) was calculated using the following equation: FB = (total fluid intake minus total fluid loss), measured in millilitres and divided by baseline body weight in kilograms. Fluid overload (FO) was defined as a cumulative fluid balance exceeding 10% of baseline body weight measured in litres [18,19].

Major adverse kidney events within a period known as MAKE-30 referred to a composite outcome occurring within 30 days post-admission or at ICU discharge based on initial patient admission criteria: mortality within 30 days and/or initiation of new renal replacement therapy (RRT), along with failure for renal function recovery within 30 days- defined as a SCr ratio comparing last reported SCr prior to Day 30 or ICU discharge against baseline SCr being equal to or greater than two 200% [20,21].

### Outcomes

The primary outcome is the occurrence of new-onset AKI, defined by its emergence in patients who experience septic shock during their admission to the ICU. Secondary outcomes include 28-day mortality and MAKE-30.

### Statistical analysis

A group-based trajectory model (GBTM) was utilized to identify subpopulations of patients exhibiting similar patterns in NE dosage progression over the initial four days of treatment. GBTM represents a specific usage of a finite mixture model designed to discern distinct populations that share similar developmental trajectories [22,23]. “Trajectory” specifically denotes the longitudinal pattern of a variable across repeated measurements, allowing the identification of groups of individuals exhibiting similar patterns over time. This methodology is based on the assumption that groups are heterogeneous and consist of a finite number of unique categories. The optimal number of trajectory clusters and the best-fitting polynomial model—linear, quadratic, or cubic—were identified using the Bayesian Information Criterion (BIC). This approach ensures the selection of a model that balances goodness-of-fit with complexity, thereby minimizing the risk of overfitting [24]. Clusters were delineated through a forward classification process, where lower BIC values indicated a more appropriate model. The required sample size must constitute at least 5% of participants, and the probability for each trajectory set should not fall below 0.70 as deemed reasonable [25]. We utilized the traj plug-in in STATA to perform GBTM for estimating NE dose trajectories [26].

Continuous variables were reported as median along with the interquartile range (IQR), while categorical variables were presented as counts and percentages. The Wilcoxon rank-sum test was used to analyze continuous variables, whereas categorical variables were evaluated using the chi-square test.

We employed Generalized Estimating Equations (GEE) to assess the relationship between NE dose trajectories and corresponding clinical outcomes. By examining the correlation structure within data, GEE facilitates efficient and unbiased estimation of regression parameters [27]. Using robust standard error estimation, we implemented the Logit link function to derive odds ratios (OR) and 95% confidence intervals (CI).

This study analyzed the independent association between NE dose trajectories and the risk of new-onset AKI through univariate and multivariate logistic regression models. Variables were entered into the model with a cutoff value of 0.1 and removed with a cutoff value of 0.05 [28]. Variance inflation factors (VIFs) and tolerance coefficients were calculated to test for multicollinearity among covariates. VIF values exceeding 10 were considered indicative of multicollinearity and were removed from the model. We established four multivariate logistic regression models with adjustments for different variables. Model I included only NE dose trajectory grouping without any adjustment; Model II adjusted for demographic characteristics (age, gender, BMI) and comorbidities (respiratory diseases, digestive diseases, diabetes) on the basis of NE dose trajectories; Model III further incorporated acute physiological status indicators (temperature, SpO2, lactate, pCO2, P/F ratio); Model IV additionally included laboratory parameters (hemoglobin, platelets, BUN, electrolytes, coagulation function, SCr) and fluid management indicators (total fluid balance) on the aforementioned basis.

In subgroup analyses involving septic shock patients, they were stratified by age, gender, BMI, diabetes, digestive disease, FO quantitative and MV. An interaction term was incorporated between stratification covariates and NE dose trajectories within fully adjusted models to assess potential effect modification. All tests were conducted bilaterally with a significance level set at 0.05. All data analyses were performed using Stata version 18.0 (Stata Corp LP, College Station, Texas), SPSS version 25.0 and R version 4.2.2.

## Results

### Baseline characteristics among latent trajectory groups

**Fig 1:**
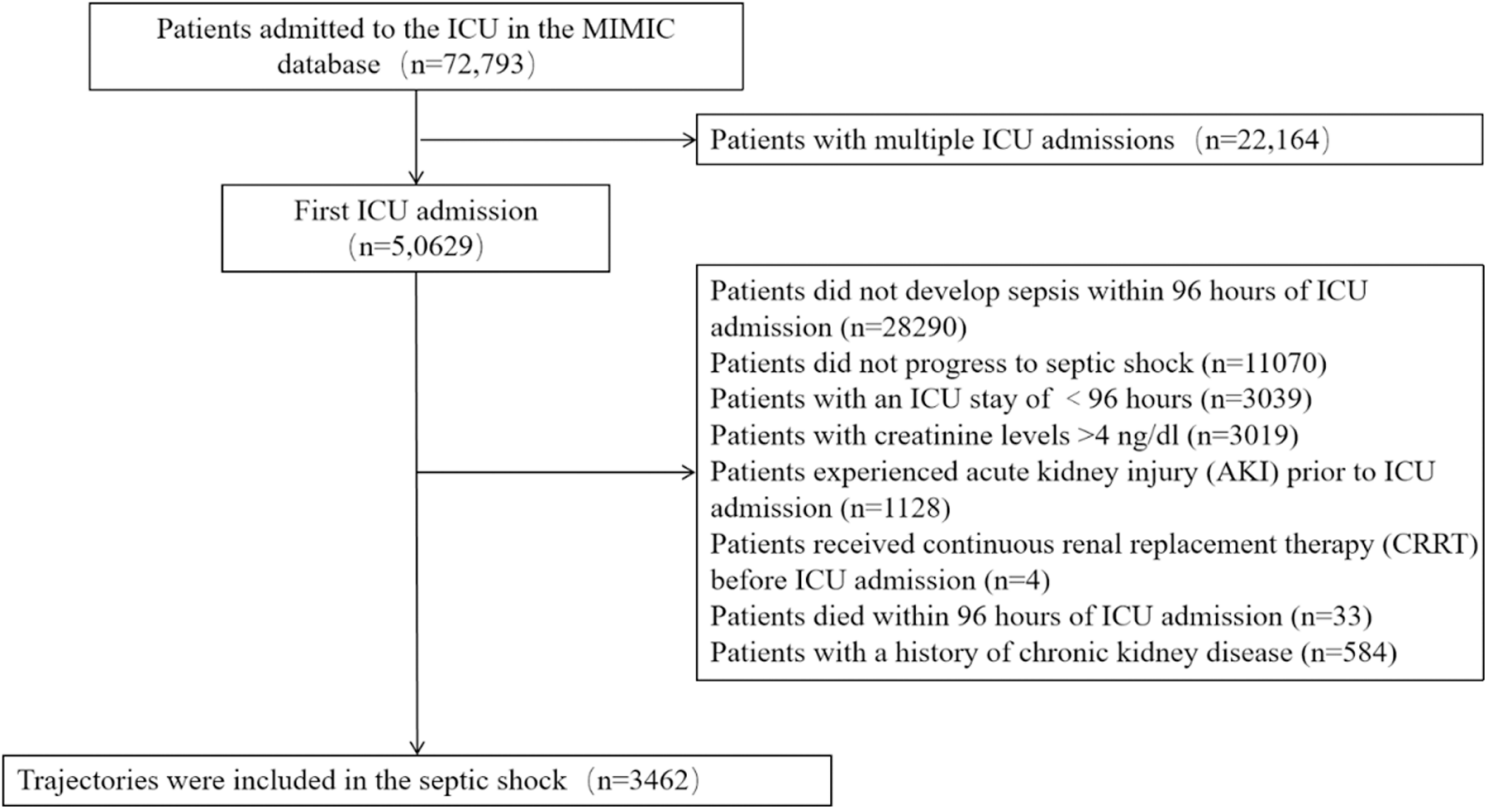
Flowchart of the patients included in the study.

A total of 72,793 critically ill patients were included in the MIMIC-IV database. After excluding 22,164 patients with multiple ICU admissions and retaining data solely from their initial admission, along with including those with a single ICU admission, 50,629 patients remained for analysis. Following an additional exclusion of 46,807 patients who failed to meet the study’s eligibility criteria, a final cohort of 3,462 patients diagnosed with septic shock within 96 hours of ICU admission and hospitalized for a minimum of 96 hours was included in the study (Fig 1).

**Fig 2:**
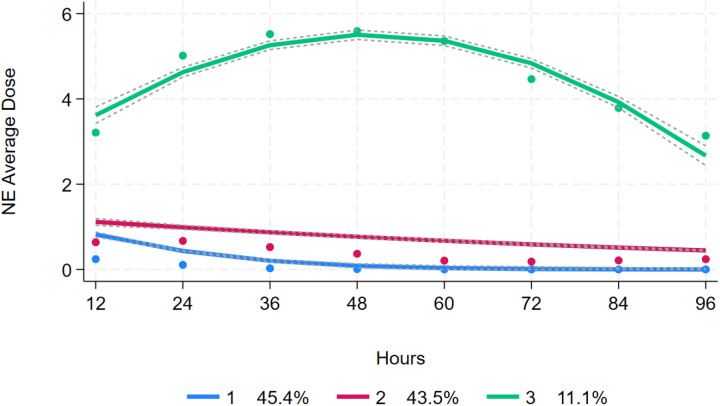
NE dose trajectory patterns during the first 4 days after admission to the ICU.

Three NE dose trajectories were identified across 8 time points (Fig 2). Trajectory 1 was classified as “low NE dose” (1,639 patients, 47.3%) and maintained the lowest average NE dose throughout the observation period while showing an initial low dose that slightly decreased over time. Trajectory 2 was characterized as “middle NE dose” (1,436 patients, 41.5%), which received a moderate average NE dose that started relatively low and showed minimal decrease while remaining stable throughout all eighttime points. Trajectory 3 was identified as the “high NE dose” group (387 patients,11.2%), receiving the highest average NE dosage that began high and gradually increased to peak around the fifth time point before declining yet remaining elevated compared to other trajectories.

At all-time points, significant differences were observed among the various NE dosage groups. The low NE dosage group received an initial dosage of 0.249 µg/kg/min at 12 hours post-admission, which gradually decreased to 0.109 µg/kg/min at 24 hours, 0.024 µg/kg/min at 36 hours and ultimately stabilized at 0.000 µg/kg/min from 48 to 96hours. The middle NE dosage group exhibited relatively stable fluctuations in dosage, starting at 0.646 µg/kg/min at 12 hours, slightly increasing to 0.694 µg/kg/min at 24 hours before subsequently decreasing to 0.252 µg/kg/min by the end of the observation period (96 hours). In contrast, the high NE dosage group reached 3.21 µg/kg/min within the first 12 hours after admission, increased further to a maximum of 5.59 µg/kg/min at 48 hours, and maintained a high level of 3.13 µg/kg/min until 96 hours (S1 Table).

In terms of cumulative dosage, the low NE dosage group exhibited a slight increase from 0.249 µg/kg/min at 12 hours to 0.391 µg/kg/min by the end of the observation period. The middle NE dosage group had a cumulative dosage of 0.646 µg/kg/min at 12 hours, which increased to 1.34 µg/kg/min at 24 hours and reached 2.67 µg/kg/min at 72 hours. Conversely, the high NE dosage group experienced rapid accumulation, rising from 3.21 µg/kg/min at 12 hours to a marked peak of 36.0 µg/kg/min at 96 hours, indicating a significant upward trend (S1 Table).

### Comparison of patient characteristics across trajectory groups

The baseline characteristics of three NE dose trajectory subtypes are shown in Table 1. When comparing demographic data, vital signs, and laboratory results across the three trajectory groups, no significant differences were observed in BMI, respiratory diseases, cancer, hematocrit, hemoglobin, calcium, chloride, sodium, fibrinogen, oxygen partial pressure (PaO2), and oxygenation index (PaO2/FiO2). However, significant differences were found in most other variables among the three groups. Notably, the high dose NE group had the highest SOFA score, showing a progressive increasing trend from the low dose to high dose groups. Simultaneously, the MAP in the high-dose group was significantly lower at 48.2 mmHg (P < 0.001). SCr levels showed a statistically significant difference among the three groups (P = 0.002), with the high-dose group exhibiting a higher trend, potentially suggesting renal function impairment in some patients in the high-dose group, although the clinical significance may be limited. Furthermore, the high-dose group had the highest fluid balance level at 49.0 (39.8–59.2) mL/kg and the highest FO level, reaching 97.4% (P < 0.001) (Table 1).

**Table 1:** Baseline characteristics of the study patients according to the 3 subgroups with different NE dose trajectory patterns.

Characteristics	ALL (N = 3462)	NE dose trajectory patterns			P-value
		Low NE group (n = 1639)	Middle NE group (n = 1436)	High NE group (n = 387)	
Demographics					
Age (years)	65 ± 16.2	64.9 ± 16.6	65.7 ± 15.8	62.6 ± 15.	0.004
Male (%)	1952 (56)	909 (55)	842 (59)	201(52)	0.036
Race (%)			0.035		
Black	254 (7.3)	126 (7.7)	96 (6.7)	32(8.3)	
White	2259 (65.3)	1082 (66.0)	950 (66.2)	227(58.7)	
Asian	94 (2.72)	51 (3.1)	34 (2.4)	9(2.3)	
Other	855 (24.7)	380 (47.3)	356 (24.8)	119(30.7)	
BMI (kg/m^2^)	28.8 (28.6–29.1)	28.6 (28.2–28.9)	28.9 (28.4–29.3)	29.4(28.6–30.2)	0.117
Comorbidities (%)^a^					
Cardiovascular disease	1753 (50.6)	814 (50.3)	761 (53.0)	178(46.0)	0.028
Respiratory disease	982 (28.4)	473 (28.9)	408 (28.4)	101(26.1)	0.555
Digestive disease	726 (21.0)	276 (16.8)	302 (21.0)	148(38.2)	< 0.001
Diabetes	913 (26.4)	276 (16.8)	302 (21.0)	148(38.2)	< 0.001
Cancer	913 (26.4)	429 (26.2)	391 (27.2)	93(24.0)	0.434
Heart rate	88 (88–89)	87 (86–88)	88(87–89)	96(94–97)	< 0.001
MAP (mmHg)	55.5 (51.2–58.8)	54.0 (52.9–55.1)	56.9 (52.9–55.1)	48.2 (47.0–49.3)	< 0.001
Respiratory rate	20 (17–23)	19 (19–20)	20 (20–21)	21 (21–22)	< 0.001
Temperature(°C)	36.9 (36.8–36.9)	36.9 (36.9–37.0)	36.8 (36.8–36.9)	37.0 (36.8–37.0)	0.007
SpO2 (%)	97 (97–97)	97(95–98)	98 (96–99)	96 (96–97)	< 0.001
Laboratory ^b^					
Hematocrit (%)	35.9 (35.7–36.2)	36.1(35.8–36.4)	35.8 (35.4–36.1)	35.8 (35.1–36.5)	0.363
Hemoglobin(g/dL)	11.8 (11.7–11.8)	11.9 (11.8–12.0)	11.7(11.6–11.8)	11.7 (11.5–11.9)	0.060
Platelets(×10³/mm³)	233 (229–238)	238 (232–244)	234 (227–240)	212 (198–224)	0.001
WBC (× 10^9^/L)	17.4 (16.9–17.8)	16.4 (15.9–16.9)	17.6 (16.8–18.4)	20.7 (19.3–22.1)	< 0.001
Bun(mg/dL)	31.2 (30.4–32.0)	28.3 (27.2–29.5)	32.9 (31.6–34.2)	36.8 (34.5–39.0)	< 0.001
Albumin(g/dL)	3.1 (3.1–3.2)	3.2 (3.1–3.2)	3.1 (3.0–3.2)	2.9 (2.8–3.0)	< 0.001
Calcium(mmol/L)	8.5 (8.5–8.6)	8.5 (8.5–8.6)	8.5 (8.5–8.6)	8.4 (8.3–8.6)	0.466
Chloride(mmol/L)	107 (107–108)	107 (107–108)	107 (107–108)	106 (106–108)	0.806
Sodium(mmol/L)	140.2 (139.9–140.3)	140.4 (140.1–140.6)	139.9 (139.9–140.2)	139.8 (139.1–140.3)	0.051
Potassium(mmol/L)	4.7 (4.7–4.8)	4.6 (4.6–4.7)	4.7 (4.7–4.8)	4.8 (4.7–5.0)	0.002
Fibrinogen(mg/dL)	333 (327–340)	333 (324–342)	331 (322–342)	339 (318–359)	0.790
INR	1.7 (1.7–1.8)	1.6(1.6–1.7)	1.8 (1.7–1.9)	2.1 (1.9–1.3)	< 0.001
PT (sec)	18.9 (18.5–19.4)	17.7 (17.1–18.2)	19.4 (18.7–20.1)	22.8 (21.1–24.4)	< 0.001
ALT(U/L)	200 (158–242)	200 (122–278)	203 (156–250)	188 (130–247)	0.015
AST(U/L)	297 (259–336)	242 (199–285)	330 (259–402)	407 (280–534)	< 0.001
SCr(mg/dL)	0.7 (0.5–0.9)	0.6 (0.5–0.9)	0.7 (0.5–0.9)	0.7 (0.5–1.0)	0.002
Lactate (mmol/L)	3.5 (3.5–3.7)	3.2 (3.0–3.3)	3.7 (3.5–3.8)	5.0 (4.6–5.4)	< 0.001
PaO2(mmHg)	249 (245–254)	250 (244–257)	250 (243–257)	243 (229–256)	0.595
PaCO2(mmHg)	49 (48–49)	49 (49–50)	48 (47–49)	50 (48–52)	0.041
PaO2/FiO2	326 (320–331)	325 (317–333)	331 (322–340)	310 (290–329)	0.085
SOFA	8 (8–8)	7 (7–8)	9 (9–9)	12 (11–12)	< 0.001
MV (%)	1061 (30.6)	417 (25.4)	475 (33.1)	169 (43.7)	< 0.001
FB (mL/kg)	22.3 (16.8–29.1)	18.2 (13.8–22.7)	25.1 (20.2–29.8)	49.0 (39.8–59.2)	< 0.001
FO (%)	1368 (39.5)	270 (16.5)	721 (50.2)	377 (97.4)	< 0.001

Data are presented as count (percent) or median (inter quartile range [IQR])

*SOFA* sequential organ failure assessment, *MV* machine ventilation, *FB* fluid balance, *FO* fluid overload

^a^Comorbidities included cardiovascular disease, respiratory disease, digestive disease, diabetes, cancer; cardiovascular disease included congestive heart failure, peripheral vascular disease, cerebrovascular disease; respiratory disease included chronic pulmonary disease; digestive disease included peptic ulcer disease, mild liver disease, severe liver disease; diabetes included diabetes with complications or comorbidities and diabetes without complications or comorbidities; cancer included metastatic solid tumor.

^b^Laboratory values were selected by the maximum values

### Characteristics of MAP dynamic changes across different NE dose trajectory groups

**Fig 3:**
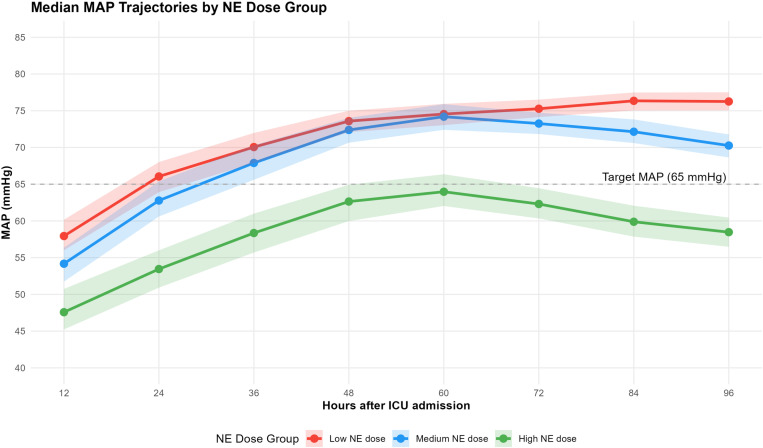
Median MAP Trajectories by NE Equivalent Dose Group During the First 96 Hours After ICU Admission.

To further understand the hemodynamic characteristics of different NE dose trajectory groups, we analyzed the dynamic changes in MAP in the three groups during the 96-hour study period, as shown in Fig 3. Combined with S2 Table, significant differences in MAP dynamics were observed among the three groups of patients during vasopressor treatment for septic shock. The low dose group's MAP started from a baseline of 57.9 mmHg, rapidly reached the hemodynamic target value (65 mmHg) at the 24-hour time point, and maintained a stable upward trend until 96 hours (76.3 mmHg). The middle dose group started from a baseline of 54.2 mmHg, delayed reaching the target until 36 hours, peaked at 60 hours, and then showed a slow decline. The high dose group presented with persistent low perfusion status, with the lowest baseline (47.6 mmHg), reaching only 64.0 mmHg at the best response point (60 hours), failing to exceed the target threshold, and subsequently deteriorating again. Differences among the three groups were statistically significant at all measurement time points (P < 0.001). Notably, despite receiving higher doses of vasopressors, the high NE dose group maintained consistently lower MAP values, at 47.6 mmHg (95% CI: 45.2–50.7) at 12 hours, and remained significantly lower than the other groups at 96 hours.

### Clinical outcomes

Table 2 presents a comprehensive summary of the clinical outcomes. Following ICU admission, 3173 patients (91.7%) developed AKI. In comparison to the low-dose NE group, the high NE group showed a markedly increased incidence of AKI (95.6% vs. 90.1%), an increased occurrence of MAKE-30 (75.5% vs. 44.6%), and elevated mortality at 28 days (44.7% vs. 19.5%). Moreover, these groups exhibited significant differences in hospital and ICU length of stay durations (P < 0.001).

**Table 2:** Clinical outcomes of the study patients with different NE dose trajectory patterns.

Characteristics	ALL (N = 3462)	NE dose trajectory patterns			P-value
**Low NE group (n = 1639)**	**Middle NE group (n = 1436)**	**High NE group (n = 387)**	
Clinical outcomes [n (%)]					
AKI	3173 (91.7)	1477 (90.1)	1326 (92.3)	370 (95.6)	< 0.001
MAKE-30 ^a^	1896 (54.8)	731 (44.6)	873 (60.8)	292 (75.5)	< 0.001
28-day mortality	961 (27.8)	319 (19.5)	469 (32.7)	173 (44.7)	< 0.001
Length of stay [d, M (IQR)]					
Hospital	19 (19–20)	18 (17–19)	21 (20–22)	22 (20–24)	< 0.001
ICU	11 (11–12)	10 (9–10)	13 (12–13)	14 (13–15)	< 0.001

*NE* norepinephrine, *AKI* acute kidney injury, *IQR* interquartile range

^a^*MAKE-30* major adverse kidney events, MAKE-30 was defined as a composite outcome of the following criteria, 30 days after admission or at ICU discharge, whichever came first: death within 30 days and/or new RRT during 30 days, and/or no renal recovery (defined as a ratio of SCr [the last recorded SCr before day 30 or ICU discharge if occurred before] to baseline SCr ≥ 200%)

### Univariate and multivariate analysis of the association between NE dose trajectory groups and AKI risk

Univariate analysis results showed that multiple factors were associated with the risk of AKI. Among these, age, BMI, respiratory disease, digestive system diseases, diabetes, temperature, SpO2, oxygen saturation, platelets, BUN, chloride, sodium, potassium ions, INR, PT, APTT, SCr, lactate, oxygenation index, FO, SOFA score, Middle NE dose group and High NE dose group demonstrated significant associations with new-onset AKI (S3 Table). Additionally, collinearity assessment using VIF indicated no significant overlap among the variables studied (S4 Table).

We present the results of a multivariate logistic analysis of the association between NE dose trajectory groups and clinical outcomes in Table 3. Across four progressive models, patients in the high NE dose group consistently exhibited a higher risk of AKI compared to the low NE dose group. In the unadjusted analysis (Model I), the high NE dose group showed a significantly increased risk of AKI (OR 2.39, 95% CI 1.43–3.99, P < 0.001). After adjusting for demographic characteristics and comorbidities (Model II), the risk in the high NE dose group slightly decreased but remained significant (OR 2.18, 95% CI 1.29–3.71, P = 0.004). With further inclusion of acute physiological status indicators (Model III), the strength of the association was somewhat attenuated (OR 1.74, 95% CI 1.03–2.94, P = 0.041). Finally, after comprehensive adjustment for all confounding factors, including laboratory indicators and fluid management (Model IV), the independent association between high NE dose trajectory and AKI remained significant (OR 1.39, 95% CI 1.04–1.86, P = 0.024). Notably, even after adjusting for baseline SCr among all confounding factors in Model IV, the independent association between high NE dose trajectory and AKI remained significant. The middle NE dose group showed a slightly increased risk of AKI in Model I (OR 1.32, 95% CI 1.02–1.70, P = 0.031), but this association was no longer significant in the adjusted model Model IV (OR 1.08, 95% CI 0.88–1.34, P = 0.419).

**Table 3:** Multivariate analysis results of the association between different NE Dose trajectory groups and clinical outcomes.

Variables	Model I		Model II		Model III		Model IV	
	**OR (95% CI)**	**P-value**	**OR (95% CI)**	**P-value**	**OR (95% CI)**	**P-value**	**OR (95% CI)**	**P-value**
Low NE	Reference		Reference		Reference		Reference	
Middle NE	1.32 (1.02–1.70)	0.031	1.23 (0.94–1.60)	0.119	1.17 (0.89–1.53)	0.240	1.08 (0.88–1.34)	0.419
High NE	2.38 (1.43–3.98)	< 0.001	2.18 (1.28–3.71)	0.004	1.75 (1.02–3.02)	0.041	1.39 (1.04–1.86)	0.024

Adjusted covariates

Model I = Three trajectory groups.

Model II = Model I + Age + BMI + Comorbidities (Respiratory disease, Digestive disease, Diabetes).

Model III = Model II + Temperature + Arterial blood gas analysis (SpO2, Lactate, PaCO2).

Model IV = Model III + Fluid balance + Laboratory examination (Hemoglobin, Platelets, BUN, Chloride, Sodium, Potassium, INR, APTT, SCr).

### Association of NE dose trajectory groups with clinical outcomes

To further evaluate the robustness of the study results, a detailed subgroup analysis was conducted based on various demographic and clinical parameters, as illustrated in Table 4. These parameters included age, gender, BMI, diabetes, digestive system diseases, FO and MV. When stratified by age, gender, BMI, diabetes, digestive system diseases, fluid status, and mechanical ventilation, the relationship between norepinephrine dosage trajectories and the incidence of newly developed AKI remained consistent. However, in comparison to the low dose trajectory, factors such as age (interaction P = 0.003), BMI (interaction P = 0.002), fluid overload (interaction P = 0.002), and mechanical ventilation (interaction P = 0.038) modified the association between the high-dose trajectory and the incidence of AKI. Additionally, age (interaction P < 0.001), BMI (interaction P < 0.001), diabetes (interaction P = 0.001) and digestive system diseases (interaction P = 0.008) altered the relationship between the middle dose trajectory and the incidence of AKI, compared to the low dose trajectory.

**Table 4:** Subgroup analysis of the associations between NE dose trajectories and AKI.

Subgroups	Events [n (%)]	Low Ref	Middle NE OR (95% CI)	*P*	*P* for interaction	High NE OR (95% CI)	*P*	*P* for interaction
**Age**								
≥65y	1912	1	1.25 (0.82–1.91)	0.295	< 0.001	3.84 (1.18–12.42)	0.025	0.003
<65y	1550	1	1.32 (0.96–1.82)	0.085		2.51 (1.38–4.57)	0.003	
**Gender**								
Male	1952	1	1.10 (0.93–1.31)	0.229	0.920	1.62 (1.14–2.30)	0.006	0.367
Female	1510	1	1.55 (1.05–2.27)	0.025		2.18 (1.11–4.31)	0.024	
**BMI**								
≤20	256	1	1.41 (0.69–2.86)	0.343	< 0.001	1.84 (0.51–6.62)	0.347	0.002
(20,25]	945	1	1.22 (0.81–1.84)	0.333		1.82 (0.87–3.80)	0.107	
> 25	2261	1	1.35 (0.93–1.95)	0.108		3.68 (1.47–9.22)	0.005	
**Diabetes**								
Yes	913	1	1.73 (0.89–3.37)	0.103	0.001	1.73 (0.89–3.37)	0.082	0.108
No	2549	1	1.23 (0.94–1.63)	0.126		2.23 (1.34–4.03)	0.003	
**Digestive disease**								
Yes	726	1	1.89 (0.90–3.94)	0.089	0.008	3.61 (1.05–12.38)	0.041	0.088
No	2736	1	1.22 (0.93–1.60)	0.143		1.95 (1.11–3.44)	0.020	
**FO**								
Yes	3263	1	2.04 (1.37–3.04)	< 0.001	0.258	5.22 (2.92–9.85)	< 0.001	0.002
No	199	1	1.49 (1.04–2.18)	0.033		0.36 (0.09–2.43)	0.204	
**MV**								
Yes	1061	1	1.59 (0.99–2.55)	0.051	0.476	6.21 (1.90–20.29)	0.002	0.038
No	2401	1	1.19 (0.88–1.61)	0.239		1.65 (0.93–2.93)	0.086	

*NE* norepinephrine, *AKI* acute kidney injury, *FO* fluid overload, *MV* mechanical ventilation

## Discussion

In this retrospective study, we employed a GBTM to characterize NE dosage patterns during the initial four days following ICU admission in patients with septic shock. Our findings indicate a correlation between NE dosage trajectories and the occurrence of new-onset AKI.

This research emphasizes the importance of longitudinal measurements of NE dosage in contrast to static single-point data or conventional mean-based grouping models. In this study, using trajectories rather than simple dose measurements or predetermined regimens allowed us to capture dynamic patterns of NE administration over time. This approach aligns with recent methodological advances in critical care research that recognize the importance of temporal variations in treatment parameters [29,30]. Trajectory analysis not only provides insights into NE dose magnitude but also encompasses its temporal evolution, rate of change, and pattern stability—dimensions that traditional single-point or mean-based assessments cannot capture. By employing a GBTM, we identified three distinct NE trajectories over the 96-hour period, highlighting the variability present among septic patients and indicating that specific clinical outcomes correlate with trends in NE dosage. Monitoring changes in NE dosage trajectories in ICU-admitted patients with septic shock is essential, as NE may exacerbate microcirculatory dysfunction and, at elevated doses, impair tissue perfusion, leading to organ dysfunction and an increased risk of AKI [31,32]. Dosage variability may be particularly pronounced in critically ill patients. Therefore, trajectory-based risk assessment represents a pivotal step toward understanding NE dosage variability in septic shock and has significant implications for current management and treatment guidelines for sepsis and septic shock. Additionally, this study innovatively employed NE equivalent dosing to standardize vasopressor support intensity. This methodology not only reflects NE utilization but also accounts for the combined effects of multiple vasopressors, enabling consistent comparison across patients receiving different pharmacological combinations and facilitating a more comprehensive assessment of the relationship between treatment intensity and clinical outcomes.

Previous research on the impact of NE dosage on clinical outcomes, including AKI and mortality, has yielded mixed results. Kotecha et al. [33] found that cumulative NE exposure is an independent predictor of mortality among critically ill patients. A retrospective study identified a correlation between greater uncorrected cumulative NE exposure, measured on a log10 scale (mg), and elevated rates of in-hospital mortality, occurrence of AKI, and one-year mortality. After adjusting for confounding variables, cumulative exposure to norepinephrine continued to serve as an independent predictor of in-hospital mortality. Ammar et al. [34] employed CART analysis to determine critical NE equivalent dose limits, facilitating the evaluation and prediction of hemodynamic stability. They identified an inverted U-shaped nonlinear relationship, suggesting that there may be an optimal NE equivalent level; levels below or above this threshold could potentially be harmful. A randomized clinical trial known as VANISH demonstrated that early administration of NE, in comparison to vasopressin, correlates with a prolonged renal failure duration in adult patients experiencing septic shock and a higher rate of renal replacement therapy utilization [35]. The aforementioned studies consistently support the association between NE use and an increased risk of adverse outcomes in critically ill patients. It is essential to acknowledge that these studies utilized static single-point measurements or conventional mean-based grouping models for analyzing NE dosage. Such methodologies may introduce bias and random errors, potentially skewing the association results while limiting the ability to fully capture the dynamic effects of NE on clinical outcomes in individuals diagnosed with sepsis.

In contrast to previous studies, our research employed continuous NE dosage data to identify distinct patterns of dosage variation over a 96-hour period and evaluate their impact on short-term clinical outcomes, particularly the incidence of new-onset AKI. This approach provides innovative insights into the temporal effects of NE dosage and its correlation with the emergence of new-onset AKI. Our findings reveal significant differences across NE dosage trajectories among patients experiencing septic shock, particularly concerning the occurrence of new-onset AKI, MAKE-30, 28-day mortality rates, duration of ICU stay, and overall length of hospitalization. The cohort characterized by high NE dosage exhibited a markedly higher incidence of new-onset AKI along with increased risks for MAKE-30 and 28-day mortality compared to the low-dosage group. Their prolonged ICU and hospital stays may reflect the added complexity and extended treatment required. Although over 90% of patients in the low-dosage group developed AKI, this cohort showed a lower risk of MAKE-30 and 28-day mortality, suggesting a more favourable prognosis concerning these outcomes.

The mechanisms underlying the relationship between NE dosage trajectories and new-onset AKI in septic shock patients remain incompletely understood. This uncertainty is partly attributable to the central role of insufficient renal perfusion and hypoxia in the pathogenesis of AKI, as well as the debated effects of varying NE dosages on renal microcirculation, blood flow distribution, and overall organ function. However, several hypotheses warrant further investigation. First, as the first-line vasopressor for managing septic shock, NE proves ineffective in approximately 17% of patients. The underlying mechanisms contributing to refractory hypotension in septic shock encompass the heightened activity of vasodilators such as nitric oxide and prostaglandins, excessive activation of ATP-sensitive potassium channels, metabolic acidosis, and an increase in inflammatory cytokines [36,37]. Collectively, these factors lead to a loss of vascular tone and diminished responsiveness to vasopressors, resulting in significant hypotension that impairs renal perfusion and may ultimately cause renal failure or mortality. Second, there appears to be an “uncoupling” between macro- and microcirculation regarding both renal and non-renal tissue responses to vasoactive drugs [38]. While NE may restore overall hemodynamic stability, it does not necessarily ameliorate microcirculatory abnormalities observed during septic shock [39]. Intravenous NE has been shown to decrease oxygenation in both the renal cortex and medulla in a dose-dependent manner [40]. Finally, NE raises arterial pressure primarily through α-adrenergic receptor-mediated vasoconstriction while slightly increasing stroke volume and cardiac output via β-adrenergic receptors [37]. However, it can also induce local renal vasoconstriction, leading to ischemia that potentially worsens renal dysfunction [41].

In this study, despite receiving the highest vasopressor support, the high NE dose group still maintained significantly lower MAP values compared to other groups, strongly suggesting that patients in the high NE dose trajectory group were in a vasopressor-resistant state rather than having inappropriate blood pressure targets. This refractory shock condition is closely associated with increased AKI risk in these patients, potentially reflecting common underlying pathophysiological processes rather than a simple causal relationship. This suggests that our observed association may more likely reflect disease severity, which simultaneously influences both NE requirements and AKI risk—a complex relationship difficult to elucidate through observational studies.

Vasopressor resistance may involve multiple mechanisms, including enhanced activity of vasodilatory substances such as nitric oxide and prostaglandins, excessive activation of ATP-sensitive potassium channels, metabolic acidosis, and increased inflammatory factors [42–44], leading to reduced vascular tone and diminished drug responsiveness. Furthermore, the inconsistency between MAP and NE dosage aligns with the phenomenon of “uncoupling” between macro- and microcirculation [45]; although high-dose NE may improve overall hemodynamics, it may not necessarily improve microcirculatory abnormalities, particularly in vital organs like the kidneys [46]. The association between high NE dosage and higher fluid balance (median 49.0 mL/kg) may reflect an insufficient response to fluid resuscitation and more severe vasodilatory status in septic shock patients, consistent with fluid balance levels reported in previous studies of severe septic shock. Despite statistically significant differences in baseline SCr values among the three dose groups (P = 0.002), median values for all groups remained within clinically normal ranges (low-dose group: 0.6 (0.5–0.9) mg/dL, middle-dose group: 0.7 (0.5–0.9) mg/dL, high-dose group: 0.7 (0.5–1.0) mg/dL). While these differences were statistically significant, their clinical significance was limited. Importantly, after adjusting for baseline SCr and other confounding factors, the association between high NE dose trajectory and new-onset AKI remained significant, indicating that this association is independent of differences in baseline renal function.

This study represents a pioneering effort to elucidate the relationships between NE dosage trajectories and the incidence of new-onset AKI, MAKE-30, and 28-day mortality in patients experiencing septic shock. Our robust analyses, supported by a large dataset, substantiate the validity of our findings. Furthermore, by utilizing high-quality clinical data from a comprehensive database, we have demonstrated a significant correlation between different NE dosage trajectory groups and adverse outcomes in septic shock patients, thereby providing new evidence for clinical decision-making.

Nevertheless, this study has several limitations. First, due to its observational design, we are unable to establish a causal relationship between norepinephrine dosage trajectories and the occurrence of AKI; therefore, caution is warranted when interpreting our findings. Second, the observational nature may leave some potential confounding factors inadequately controlled, which could influence the observed associations. Third, we did not account for the use of vasopressors or vasoactive drugs prior to ICU admission or during surgery, which represents a crucial consideration. Future analyses incorporating these variables will enhance our understanding of the association between norepinephrine dosage trajectories and the risks of progressive chronic kidney disease and end-stage renal disease. Additionally, further investigations involving larger sample sizes are necessary to corroborate our results.

## Conclusion

In critically ill patients with septic shock, distinct trajectories of norepinephrine dosage are significantly associated with clinical outcomes. An increase in norepinephrine dosage is correlated with higher incidences of new-onset AKI, MAKE-30, and an elevated risk of 28-day mortality. Furthermore, an independent association was demonstrated between high NE dose trajectories and the development of new-onset AKI, providing novel insights for identifying high-risk patients in clinical practice. This finding necessitates confirmation through additional prospective clinical studies.

## Supporting information

S1 Table Daily norepinephrine dose and cumulative norepinephrine dose in the 3 subgroups with different trajectory patterns. (DOCX)

S2 Table Temporal changes in MAP among different NE dose trajectory groups within 96 hours after ICU admission. (DOCX)

S3 Table Univariate logistic regression analysis of risk factors associated with new-set AKI. (DOCX)

S4 Table Collinearity analysis. (DOCX)

S5 Dataset. Available data. (XLSV)

## References

[1] Uchino S, Kellum JA, Bellomo R, Doig GS, Morimatsu H, Morgera S, et al. Acute renal failure in critically ill patients: a multinational, multicenter study. JAMA. 2005;294(7):813–8. doi: 10.1001/jama.294.7.813 16106006

[2] Hoste EAJ, Kellum JA, Selby NM, Zarbock A, Palevsky PM, Bagshaw SM, et al. Global epidemiology and outcomes of acute kidney injury. Nat Rev Nephrol. 2018;14(10):607–25. doi: 10.1038/s41581-018-0052-0 30135570

[3] Singer M, Deutschman CS, Seymour CW, Shankar-Hari M, Annane D, Bauer M, et al. The Third International Consensus Definitions for Sepsis and Septic Shock (Sepsis-3). JAMA. 2016;315(8):801–10. doi: 10.1001/jama.2016.0287 26903338 PMC4968574

[4] Scheeren TWL, Bakker J, De Backer D, Annane D, Asfar P, Boerma EC, et al. Current use of vasopressors in septic shock. Ann Intensive Care. 2019;9(1):20. doi: 10.1186/s13613-019-0498-7 30701448 PMC6353977

[5] Rhodes A, Evans LE, Alhazzani W, Levy MM, Antonelli M, Ferrer R, et al. Surviving Sepsis Campaign: International Guidelines for Management of Sepsis and Septic Shock: 2016. Intensive Care Med. 2017;43(3):304–77. doi: 10.1007/s00134-017-4683-6 28101605

[6] Döpp-Zemel D, Groeneveld ABJ. High-dose norepinephrine treatment: determinants of mortality and futility in critically ill patients. Am J Crit Care. 2013;22(1):22–32. doi: 10.4037/ajcc2013748 23283085

[7] Brand DA, Patrick PA, Berger JT, Ibrahim M, Matela A, Upadhyay S, et al. Intensity of Vasopressor Therapy for Septic Shock and the Risk of In-Hospital Death. J Pain Symptom Manage. 2017;53(5):938–43. doi: 10.1016/j.jpainsymman.2016.12.333 28062334

[8] Hollenberg SM. Vasopressor support in septic shock. Chest. 2007;132(5):1678–87. doi: 10.1378/chest.07-0291 17998371

[9] Auchet T, Regnier M-A, Girerd N, Levy B. Outcome of patients with septic shock and high-dose vasopressor therapy. Ann Intensive Care. 2017;7(1):43. doi: 10.1186/s13613-017-0261-x 28425079 PMC5397393

[10] Johnson A, Bulgarelli L, Shen L, Gayles A, Shammout A, Horng S. MIMIC-IV, a freely accessible electronic health record dataset. Sci Data. 2023;10:1. 36596836 10.1038/s41597-022-01899-x PMC9810617

[11] Vincent JL, de Mendonça A, Cantraine F, Moreno R, Takala J, Suter PM, et al. Use of the SOFA score to assess the incidence of organ dysfunction/failure in intensive care units: results of a multicenter, prospective study. Working group on “sepsis-related problems” of the European Society of Intensive Care Medicine. Crit Care Med. 1998;26: 1793–800. 10.1097/00003246-199811000-00016 9824069

[12] Gong K, Lee HK, Yu K, Xie X, Li J. A prediction and interpretation framework of acute kidney injury in critical care. J Biomed Inform. 2021;113:103653. doi: 10.1016/j.jbi.2020.103653 33338667

[13] Sterne JAC, White IR, Carlin JB, Spratt M, Royston P, Kenward MG, et al. Multiple imputation for missing data in epidemiological and clinical research: potential and pitfalls. BMJ. 2009;338:b2393. doi: 10.1136/bmj.b2393 19564179 PMC2714692

[14] Jentzer JC, Vallabhajosyula S, Khanna AK, Chawla LS, Busse LW, Kashani KB. Management of Refractory Vasodilatory Shock. Chest. 2018;154(2):416–26. doi: 10.1016/j.chest.2017.12.021 29329694

[15] Shankar-Hari M, Phillips GS, Levy ML, Seymour CW, Liu VX, Deutschman CS, et al. Developing a New Definition and Assessing New Clinical Criteria for Septic Shock: For the Third International Consensus Definitions for Sepsis and Septic Shock (Sepsis-3). JAMA. 2016;315(8):775–87. doi: 10.1001/jama.2016.0289 26903336 PMC4910392

[16] Khwaja A. KDIGO clinical practice guidelines for acute kidney injury. Nephron Clin Pract. 2012;120(4):c179-84. doi: 10.1159/000339789 22890468

[17] Ronco C, Bellomo R, Kellum JA. Acute kidney injury. Lancet. 2019;394(10212):1949–64. doi: 10.1016/S0140-6736(19)32563-2 31777389

[18] Fülöp T, Pathak MB, Schmidt DW, Lengvárszky Z, Juncos JP, Lebrun CJ, et al. Volume-related weight gain and subsequent mortality in acute renal failure patients treated with continuous renal replacement therapy. ASAIO J. 2010;56(4):333–7. doi: 10.1097/MAT.0b013e3181de35e4 20559136 PMC2895683

[19] Wang M-P, Jiang L, Zhu B, Du B, Li W, He Y, et al. Association of fluid balance trajectories with clinical outcomes in patients with septic shock: a prospective multicenter cohort study. Mil Med Res. 2021;8(1):40. doi: 10.1186/s40779-021-00328-1 34225807 PMC8258941

[20] McKown AC, Wang L, Wanderer JP, Ehrenfeld J, Rice TW, Bernard GR, et al. Predicting Major Adverse Kidney Events among Critically Ill Adults Using the Electronic Health Record. Med Syst. 2017;41: 1–7. 10.1007/s10916-017-0806-4 PMC5821255 28861688

[21] Semler MW, Rice TW, Shaw AD, Siew ED, Self WH, Kumar AB, et al. Identification of Major Adverse Kidney Events Within the Electronic Health Record. J Med Syst. 2016;40: 1–10. 27234478 10.1007/s10916-016-0528-z PMC5791539

[22] Nagin DS, Odgers CL. Group-based trajectory modeling in clinical research. Annu Rev Clin Psychol. 2010;6:109–38. doi: 10.1146/annurev.clinpsy.121208.131413 20192788

[23] Nagin D. Group-based modeling of development. Harvard University Press. 2005.

[24] Nagin D, Tremblay R. Analyzing developmental trajectories of distinct but related behaviors: a group-based method. Psychol Methods. 2001;6:18. 11285809 10.1037/1082-989x.6.1.18

[25] Schwandt A, Hermann JM, Rosenbauer J, Boettcher C, Dunstheimer D, Grulich-Henn J, et al. Longitudinal Trajectories of Metabolic Control From Childhood to Young Adulthood in Type 1 Diabetes From a Large German/Austrian Registry: A Group-Based Modeling Approach. Diabetes Care. 2017;40(3):309–16. doi: 10.2337/dc16-1625 28007778

[26] Jones L, Nagin D. A Stata plugin for estimating group-based trajectory models.

[27] Liang K, Zeger S. Longitudinal data analysis using generalized linear models. Biometrics. 1986;73:13–22.

[28] VanderWeele TJ. Principles of confounder selection. Eur J Epidemiol. 2019;34(3):211–9. doi: 10.1007/s10654-019-00494-6 30840181 PMC6447501

[29] Deng B, Liu Q, Qiao L, Lv S. Longitudinal trajectories of blood glucose and 30-day mortality in patients with diabetes mellitus combined with acute myocardial infarction: A retrospective cohort analysis of the MIMIC database. PLoS One. 2024;19(9):e0307905. doi: 10.1371/journal.pone.0307905 39269943 PMC11398677

[30] Yang J, Ma B, Tong H. Lymphocyte count trajectories are associated with the prognosis of sepsis patients. Crit Care. 2024;28(1):399. doi: 10.1186/s13054-024-05186-6 39623501 PMC11613510

[31] Lankadeva YR, Kosaka J, Evans RG, Bailey SR, Bellomo R, May CN. Intrarenal and urinary oxygenation during norepinephrine resuscitation in ovine septic acute kidney injury. Kidney Int. 2016;90: 100–8. 27165831 10.1016/j.kint.2016.02.017

[32] Lankadeva YR, Ma S, Iguchi N, Evans RG, Hood SG, Farmer DGS, et al. Dexmedetomidine reduces norepinephrine requirements and preserves renal oxygenation and function in ovine septic acute kidney injury. Kidney Int. 2019;96(5):1150–61. doi: 10.1016/j.kint.2019.06.013 31530477

[33] Kotecha AA, Vallabhajosyula S, Apala DR, Frazee E, Iyer VN. Clinical Outcomes of Weight-Based Norepinephrine Dosing in Underweight and Morbidly Obese Patients: A Propensity-Matched Analysis. J Intensive Care Med. 2020;35(6):554–61. doi: 10.1177/0885066618768180 29628015

[34] Ammar MA, Limberg EC, Lam SW, Ammar AA, Sacha GL, Reddy AJ, et al. Optimal norepinephrine-equivalent dose to initiate epinephrine in patients with septic shock. J Crit Care. 2019;53:69–74. doi: 10.1016/j.jcrc.2019.05.024 31202160

[35] Gordon AC, Mason AJ, Thirunavukkarasu N, Perkins GD, Cecconi M, Cepkova M, et al. Effect of Early Vasopressin vs Norepinephrine on Kidney Failure in Patients With Septic Shock: The VANISH Randomized Clinical Trial. JAMA. 2016;316(5):509–18. doi: 10.1001/jama.2016.10485 27483065

[36] Kimmoun A, Ducrocq N, Levy B. Mechanisms of vascular hyporesponsiveness in septic shock. Curr Vasc Pharmacol. 2013;11:139–49. 23506493

[37] Pollard S, Edwin SB, Alaniz C. Vasopressor and Inotropic Management Of Patients With Septic Shock. P T. 2015;40(7):438–50. 26185405 PMC4495871

[38] Ma S, Evans RG, Iguchi N, Tare M, Parkington HC, Bellomo R, et al. Sepsis-induced acute kidney injury: A disease of the microcirculation. Microcirculation. 2019;26(2):e12483. doi: 10.1111/micc.12483 29908046

[39] Krejci V, Hiltebrand L, Sigurdsson G. Effects of epinephrine, norepinephrine, and phenylephrine on microcirculatory blood flow in the gastrointestinal tract in sepsis. Crit Care Med. 2006;34:1456–63. 16557162 10.1097/01.CCM.0000215834.48023.57

[40] Langenberg C, Bagshaw SM, May CN, Bellomo R. The histopathology of septic acute kidney injury: a systematic review. Crit Care. 2008;12(2):R38. doi: 10.1186/cc6823 18325092 PMC2447560

[41] Bellomo R, Wan L, May C. Vasoactive drugs and acute kidney injury. Crit Care Med. 2008;36(4 Suppl):S179-86. doi: 10.1097/CCM.0b013e318169167f 18382191

[42] Manoury B, Idres S, Leblais V, Fischmeister R. Ion channels as effectors of cyclic nucleotide pathways: Functional relevance for arterial tone regulation. Pharmacol Ther. 2020;209:107499. doi: 10.1016/j.pharmthera.2020.107499 32068004

[43] Tykocki NR, Boerman EM, Jackson WF. Smooth Muscle Ion Channels and Regulation of Vascular Tone in Resistance Arteries and Arterioles. Compr Physiol. 2017;7(2):485–581. doi: 10.1002/cphy.c160011 28333380 PMC5575875

[44] Barrett LK, Singer M, Clapp LH. Vasopressin: mechanisms of action on the vasculature in health and in septic shock. Crit Care Med. 2007;35(1):33–40. doi: 10.1097/01.CCM.0000251127.45385.CD 17133186

[45] Fatnassi A, Romdhani C, Kaabi W, Labbene I, Hajjej Z, Sellami W, et al. Microcirculatory effects of norepinephrine in patients with septic shock: a microdialysis study. Research Square Platform LLC. 2020. doi: 10.21203/rs.3.rs-17259/v1

[46] Lankadeva YR, Okazaki N, Evans RG, Bellomo R, May CN. Renal Medullary Hypoxia: A New Therapeutic Target for Septic Acute Kidney Injury?. Semin Nephrol. 2019;39(6):543–53. doi: 10.1016/j.semnephrol.2019.10.004 31836037

