# Supplementary material for: High NE dose trajectory is associated with new onset of acute kidney injury patients: A group-based trajectory modeling analysis
Source: PLoS One. 2025 May 13;20(5):e0323431. doi: 10.1371/journal.pone.0323431 (PMC12074548; doi:10.1371/journal.pone.0323431)
Supplement: S1 Table — (DOCX) [file pone.0323431.s001.docx]

**Supplementary Information**

**S1 Table. Daily norepinephrine dose and cumulative norepinephrine dose in the 3 subgroups with different trajectory patterns.**

| **Time(hour)** | **NE dose trajectory** | **NE Mean Dose (Cumulative Dose)** | | **Sample Size (n)** |
| --- | --- | --- | --- | --- |
| 12 | Low NE dose | 0.249(0.249) | 308 | |
| 12 | Middle NE dose | 0.646(0.646) | 2952 | |
| 12 | High NE dose | 3.21(3.21) | 202 | |
| 24 | Low NE dose | 0.109(0.358) | 308 | |
| 24 | Middle NE dose | 0.694(1.34) | 2952 | |
| 24 | High NE dose | 5.01(8.21) | 2102 | |
| 36 | Low NE dose | 0.024(0.382) | 308 | |
| 36 | Middle NE dose | 0.548(1.89) | 2952 | |
| 36 | High NE dose | 5.51(13.7) | 202 | |
| 48 | Low NE dose | 0.007(0.390) | 308 | |
| 48 | Middle NE dose | 0.381(2.27) | 2952 | |
| 48 | High NE dose | 5.59(24.7) | 202 | |
| 60 | Low NE dose | 0.000(0.391) | 308 | |
| 60 | Middle NE dose | 0.21(2.48) | 2952 | |
| 60 | High NE dose | 5.37(29.1) | 202 | |
| 72 | Low NE dose | 0.000(0.391) | 308 | |
| 72 | Middle NE dose | 0.193(2.67) | 2952 | |
| 72 | High NE dose | 4.45(32.9) | 202 | |
| 84 | Low NE dose | 0.000(0.391) | 308 | |
| 84 | Middle NE dose | 0.221(2.89) | 2952 | |
| 84 | High NE dose | 3.78(32.9) | 202 | |
| 96 | Low NE dose | 0.000(0.391) | 308 | |
| 96 | Middle NE dose | 0.252(3.14) | 2952 | |
| 96 | High NE dose | 3.13(36.0) | 202 | |
