# Supplementary material for: High NE dose trajectory is associated with new onset of acute kidney injury patients: A group-based trajectory modeling analysis
Source: PLoS One. 2025 May 13;20(5):e0323431. doi: 10.1371/journal.pone.0323431 (PMC12074548; doi:10.1371/journal.pone.0323431)
Supplement: S2 Table — (DOCX) [file pone.0323431.s002.docx]

**S2 Table. Temporal Changes in MAP among different NE dose trajectory groups within 96 hours after ICU admission**

| **Time (hours)** | **Low NE group** | **Middle NE group** | **High NE group** | **P value** |
| --- | --- | --- | --- | --- |
| 12 | 57.9 (56.0-60.2) | 54.2 (51.7-56.3) | 47.6 (45.2-50.7) | <0.001 |
| 24 | 66.1 (63.9-68.0) | 62.8 (60.6-65.4) | 53.4 (50.9-56.0) | <0.001 |
| 36 | 70.1 (67.9-72.0) | 67.9 (65.6-70.3) | 58.4 (55.7-61.0) | <0.001 |
| 48 | 73.6 (72.1-75.0) | 72.4 (70.6-74.0) | 62.6 (60.0-64.9) | <0.001 |
| 60 | 74.6 (73.0-75.9) | 74.2 (72.4-75.9) | 64.0 (62.0-66.4) | <0.001 |
| 72 | 75.3 (74.1-76.5) | 73.3 (71.8-74.7) | 62.3 (60.3-64.5) | <0.001 |
| 84 | 76.4 (75.0-77.5) | 72.1 (70.6-73.8) | 59.9 (57.9-62.1) | <0.001 |
| 96 | 76.3 (75.1-77.5) | 70.3 (68.6-71.8) | 58.5 (56.5-60.5) | <0.001 |
