# Supplementary material for: High NE dose trajectory is associated with new onset of acute kidney injury patients: A group-based trajectory modeling analysis
Source: PLoS One. 2025 May 13;20(5):e0323431. doi: 10.1371/journal.pone.0323431 (PMC12074548; doi:10.1371/journal.pone.0323431)
Supplement: S3 Table — (DOCX) [file pone.0323431.s003.docx]

**S3 Table. Univariate logistic regression analysis of risk factors associated with new-set AKI**

| **Variables** | **β** | **S. E.** | **Z** | **OR (95%CI)** | **P value** |
| --- | --- | --- | --- | --- | --- |
| Age | 0.03 | 0.00 | 8.59 | 1.03(1.02-1.04) | < 0.001 |
| Gender |  |  |  |  |  |
| 1 |  |  |  |  |  |
| 2 | 0.12 | 0.12 | 0.98 | 1.13(0.89-1.44) | 0.325 |
| BMI (kg/m²) | 0.11 | 0.01 | 9.27 | 1.12(1.09-1.14) | < 0.001 |
| Cardiovascular disease |  |  |  |  |  |
| 0 |  |  |  |  |  |
| 1 | 0.17 | 0.12 | 1.39 | 1.19(0.93-1.51) | 0.164 |
| Respiratory disease |  |  |  |  |  |
| 0 |  |  |  |  |  |
| 1 | 0.56 | 0.15 | 3.64 | 1.76(1.30-2.38) | < 0.001 |
| Digestive disease |  |  |  |  |  |
| 0 |  |  |  |  |  |
| 1 | 0.70 | 0.19 | 3.79 | 2.02(1.40-2.91) | < 0.001 |
| Diabetes |  |  |  |  |  |
| 0 |  |  |  |  |  |
| 1 | 0.83 | 0.17 | 4.79 | 2.29(1.63-3.22) | < 0.001 |
| Cancer |  |  |  |  |  |
| 0 |  |  |  |  |  |
| 1 | 0.22 | 0.28 | 0.79 | 1.25(0.72-2.18) | 0.430 |
| Heart rate | 0.00 | 0.00 | 0.05 | 1.00(0.99-1.01) | 0.961 |
| Respiratory rate | 0.00 | 0.01 | 0.03 | 1.00(0.97-1.03) | 0.973 |
| Temperature | -0.45 | 0.09 | -4.77 | 0.64(0.53-0.77) | < 0.001 |
| SpO2 | -0.19 | 0.04 | -5.50 | 0.82(0.77-0.88) | < 0.001 |
| MAP | -0.01 | 0.02 | -0.73 | 0.99(0.95-1.02) | 0.462 |
| Hematocrit | -0.01 | 0.01 | -0.64 | 0.99(0.98-1.01) | 0.522 |
| Hemoglobin | -0.06 | 0.03 | -2.13 | 0.94(0.89-1.00) | 0.034 |
| Platelets | 0.00 | 0.00 | -3.14 | 1.00(1.00-1.00) | 0.002 |
| WBC | 0.00 | 0.01 | 0.45 | 1.00(0.99-1.01) | 0.651 |
| BUN | 0.01 | 0.00 | 4.14 | 1.01(1.01-1.02) | < 0.001 |
| Albumin | -0.13 | 0.09 | -1.53 | 0.88(0.74-1.04) | 0.126 |
| Calcium | 0.13 | 0.07 | 1.85 | 1.14(0.99-1.31) | 0.065 |
| Chloride | -0.03 | 0.01 | -3.49 | 0.97(0.95-0.99) | < 0.001 |
| Sodium | -0.04 | 0.01 | -3.62 | 0.96(0.94-0.98) | < 0.001 |
| Potassium | 0.37 | 0.08 | 4.39 | 1.44(1.23-1.70) | < 0.001 |
| Fibrinogen | 0.00 | 0.00 | 0.13 | 1.00(1.00-1.00) | 0.900 |
| INR | 1.05 | 0.17 | 6.36 | 2.86(2.07-3.96) | < 0.001 |
| PT | 0.10 | 0.02 | 6.31 | 1.10(1.07-1.14) | < 0.001 |
| APTT | 0.01 | 0.00 | 3.86 | 1.01(1.00-1.01) | < 0.001 |
| ALT | 0.00 | 0.00 | 0.50 | 1.00(1.00-1.00) | 0.616 |
| AST | 0.00 | 0.00 | 1.73 | 1.00(1.00-1.00) | 0.084 |
| SCr | 2.22 | 0.25 | 8.94 | 9.25(5.68-15.06) | < 0.001 |
| Lactate | 0.13 | 0.03 | 4.46 | 1.14(1.07-1.20) | < 0.001 |
| PaO2 | 0.00 | 0.00 | -0.67 | 1.00(1.00-1.00) | 0.502 |
| PaCO2 | 0.01 | 0.00 | 2.08 | 1.01(1.00-1.02) | 0.037 |
| PaO2/FiO2 ratio | 0.00 | 0.00 | -5.08 | 1.00(1.00-1.00) | < 0.001 |
| SOFA | 0.16 | 0.02 | 8.71 | 1.17(1.13-1.21) | < 0.001 |
| MV |  |  |  |  |  |
| 0 |  |  |  |  |  |
| 1 | 0.14 | 0.14 | 1.01 | 1.15(0.88-1.50) | 0.313 |
| Low NE |  |  |  |  |  |
| Middle NE | 0.28 | 0.13 | 2.16 | 1.32(1.03-1.70) | 0.031 |
| High NE | 0.87 | 0.26 | 3.33 | 2.39(1.43-3.99) | < 0.001 |
| FB | 0.00 | 0.00 | 4.90 | 1.00(1.00-1.00) | < 0.001 |
| FO |  |  |  |  |  |
| 0 |  |  |  |  |  |
| 1 | -0.28 | 0.12 | -2.23 | 0.76(0.60-0.97) | 0.026 |

**Abbreviations**: AKI: Acute Kidney Injury; ALT: Alanine Aminotransferase; APTT: Activated Partial Thromboplastin Time; AST: Aspartate Aminotransferase; BMI: Body Mass Index; BUN: Blood Urea Nitrogen; CI: Confidence Interval; FB: Fluid Balance; FiO2: Fraction of Inspired Oxygen; FO: Fluid Overload; INR: International Normalized Ratio; MAP: Mean Arterial Pressure; MV: Mechanical Ventilation; NE: Norepinephrine; OR: Odds Ratio; PaCO2: Partial Pressure of Carbon Dioxide; PaO2: Partial Pressure of Oxygen; PT: Prothrombin Time; S.E.: Standard Error; SCr: Serum Creatinine; SOFA: Sequential Organ Failure Assessment; SpO2: Peripheral Oxygen Saturation; WBC: White Blood Cell count.
