# Supplementary material for: High NE dose trajectory is associated with new onset of acute kidney injury patients: A group-based trajectory modeling analysis
Source: PLoS One. 2025 May 13;20(5):e0323431. doi: 10.1371/journal.pone.0323431 (PMC12074548; doi:10.1371/journal.pone.0323431)
Supplement: S4 Table — (DOCX) [file pone.0323431.s004.docx]

**S4 Table. Collinearity analysis**

| **Variables** | **P-value** | **Variance Inflation Factor** | | **Tolerance** |
| --- | --- | --- | --- | --- |
| Sodium | 0.376 | 1.706 | 0.585 | |
| Chloride | 0.895 | 1.704 | 0.586 | |
| FB | < 0.001 | 1.510 | 0.661 | |
| NE trajectory | 0.163 | 1.223 | 0.817 | |
| BUN | 0.092 | 1.153 | 0.867 | |
| PaO2/FiO2 ratio | 0.074 | 1.132 | 0.882 | |
| SpO2 | 0.021 | 1.129 | 0.885 | |
| SCr | < 0.001 | 1.108 | 0.901 | |
| Age | < 0.001 | 1.108 | 0.902 | |
| Potassium | 0.109 | 1.072 | 0.932 | |
| PaCO2 | 0.934 | 1.071 | 0.933 | |
| Hemoglobin | 0.266 | 1.066 | 0.937 | |
| Lactate | 0.011 | 1.062 | 0.940 | |
| Temperature | 0.002 | 1.062 | 0.941 | |
| BMI | < 0.001 | 1.054 | 0.948 | |
| Digestive disease | 0.033 | 1.052 | 0.950 | |
| Respiratory disease | 0.062 | 1.049 | 0.952 | |
| Diabetes | 0.405 | 1.037 | 0.964 | |
| APTT | 0.078 | 1.026 | 0.974 | |
| Platelets | 0.074 | 1.025 | 0.975 | |

Abbreviations：AKI: Acute Kidney Injury；ALT: Alanine Aminotransferase; APTT: Activated Partial Thromboplastin Time; AST: Aspartate Aminotransferase; BMI: Body Mass Index; BUN: Blood Urea Nitrogen; CI: Confidence Interval; FB: Fluid Balance; FiO2: Fraction of Inspired Oxygen; FO: Fluid Overload; INR: International Normalized Ratio; MAP: Mean Arterial Pressure; MV: Mechanical Ventilation; NE: Norepinephrine; OR: Odds Ratio; PaCO2: Partial Pressure of Carbon Dioxide; PaO2: Partial Pressure of Oxygen; PT: Prothrombin Time; S.E.: Standard Error; SCr: Serum Creatinine; SOFA: Sequential Organ Failure Assessment; SpO2: Peripheral Oxygen Saturation; WBC: White Blood Cell count.
